# Anti-Bacterial and Anti-Candidal Activity of Silver Nanoparticles Biosynthesized Using *Citrobacter* spp. MS5 Culture Supernatant

**DOI:** 10.3390/biom10060944

**Published:** 2020-06-22

**Authors:** Aftab Hossain Mondal, Dhananjay Yadav, Asghar Ali, Neelofar Khan, Jun O Jin, Qazi Mohd Rizwanul Haq

**Affiliations:** 1 Department of Biosciences, Jamia Millia Islamia, New Delhi 110025, India; aftabmicro@gmail.com (A.H.M.); asg.bstlko@gmail.com (A.A.); khannilofarkhan@gmail.com (N.K.); 2 Department of Medical Biotechnology, Yeungnam University, Gyeongsan 712-749, Korea; dhanyadav16481@gmail.com; 3 Research Institute of Cell Culture, Yeungnam University, Gyeongsan 38541, Korea

**Keywords:** silver nanoparticles, *Citrobacter* spp. MS5, Gram-negative bacteria, antibacterial, antifungal

## Abstract

The present study described the extracellular synthesis of silver nanoparticles (AgNPs) using environmental bacterial isolate *Citrobacter* spp. MS5 culture supernatant. To our best knowledge, no previous study reported the biosynthesis of AgNPs using this bacterial isolate. The biosynthesized AgNPs were characterized using different techniques like UV-Vis spectroscopy, fourier transform infrared spectroscopy (FTIR), X-ray diffraction (XRD), transmission electron microscopy (TEM), scanning electron microscopy (SEM) equipped with energy dispersive X-ray (EDX). The analysis of UV-Vis spectra revealed absorption maxima at 415 nm due to surface plasmon resonance (SPR) indicated the formation of AgNPs and FTIR spectrum confirmed the participation of proteins molecule in AgNPs synthesis. XRD and EDX spectrum confirmed the metallic and crystalline nature of AgNPs. TEM and SEM showed spherical nanoparticles with a size range of 5–15 nm. The biosynthesized AgNPs showed effective independent as well as enhanced combined antibacterial activity against extended spectrum β-lactamase (ESBL) producing multidrug resistant Gram-negative bacteria. Further, effective antifungal activity of AgNPs was observed towards pathogenic *Candida* spp. The present study provides evidence for eco-friendly biosynthesis of well-characterized AgNPs and their potential antibacterial as well as antifungal activity.

## 1. Introduction

Nanotechnology deals with design, fabrication, production, and application of 1–100 nm size of particles. Unlike bulk materials, nanoparticles exhibited unique size and shape-dependent properties including physical, chemical, and biological in different scientific fields [1,2]. There are various categories of metallic nanoparticles such as silver, gold, zinc, and iron, etc. Among them, silver nanoparticles (AgNPs) have been widely studied in biological fields for use as antibacterial, antifungal, anti-cancer, drug delivery, biomolecular detection, and catalysis [3,4]. Among the various applications, AgNPs are well known for their antibacterial activity but very few studies reported its efficacy against extended spectrum β-lactamase (ESBL) producing bacteria [5,6]. The ESBLs are a group of β-lactamases which have the ability to hydrolyse various types of antibiotics including penicillins, up to third generation cephalosporins (cefotaxime, ceftriaxone, ceftazidime) and monobactams (aztreonam) [7]. The ESBLs producing bacteria are not only restricted in clinical settings but also arise in an aquatic environment [8,9]. Generally, river aquatic ecosystem encourages antibiotic resistant bacteria to exchange their resistance genes to other sensitive bacteria by horizontal gene transfer (HGT) to create antibiotic resistant human pathogens. The increasing prevalence of ESBLs producing bacteria creates great health care problems throughout the world, therefore there is a current need to search alternative therapy to prevent them [10,11]. Studies also reported that various fungal infections and their associated mortality rate have dramatically increased in the population. Among fungus, *Candida* spp. is the most common pathogen, which is accountable for most of the infections [12]. The increasing morbidity and mortality due to infection caused by pathogenic microbes oblige scientific community to develop novel compounds with new target sites to prevent them. In order to promote alternative strategies towards combating microorganisms, AgNPs could be a potent antimicrobial agent.

Due to the physiochemical properties and wide applications of metal nanoparticles in the diverse branches of sciences and technology, the scientific community has devoted extensive efforts to develop suitable techniques for well-characterized nanoparticles synthesis. Mainly three methods including physical, chemical, and biological are used for the synthesis of nanoparticles [13]. However, physical and chemical methods are most popular to produce well-characterized nanoparticles, but use of enormous energy and production of toxic byproducts make them ‘not a preferable’ approach [14,15]. The traditional and most popular way for metal nanoparticles synthesis is the chemical method. Usually, chemical method is simple and required low cost but have some drawbacks like use of toxic solvent, unwanted by-products, and contamination from precursor chemicals. Therefore, the development of green synthesis of nanoparticles is an emerging demand to avoid conventional hazard of chemical toxicity. Consequently, biological methods take place as an important route for nanoparticles synthesis due to its cost-effective, non-toxic, and eco-friendly properties [16,17]. For this purpose, we have myriad numbers of biological resources available in natural systems such as plants or plant products, microorganisms such as bacteria, algae, and fungi [14,17,18]. Among the microorganisms, bacteria are mostly chosen for synthesis of nanoparticles due to their growing success, easy handling, and achievable genetic manipulation [19,20]. Bacteria can synthesize nanoparticles by both intracellular as well as extracellular approaches, as per the location where metallic nanoparticles are synthesized. The intracellular process consists of entry of metal ions from environmental into the microbes to synthesize nanoparticles using various enzymes. While extracellular synthesis of nanoparticles includes deceiving the respective metal ions using various enzymes present on the surface of microbes [21]. However, the extracellular approach is mostly used to understand the mechanism of synthesis, rapid scale-up, and simple isolation of nanoparticles.

In this study, we have investigated the extracellular synthesis of AgNPs using *Citrobacter* spp. MS5 culture supernatant. To the best of our knowledge, extracellular biosynthesis of AgNPs using this bacterial isolate has not been reported so far. Furthermore, we evaluated the antimicrobial activity of biosynthesized AgNPs against ESBL producing multidrug-resistant Gram-negative bacteria and pathogenic *Candida* spp.

## 2. Methodology

### 2.1. Materials

All the microbiological culture media and antibiotics were purchased from Himedia, India. Silver nitrate (AgNO_3_) with 99% purity was obtained from Merck Ltd., India. Sterile Milli-Q water and glassware were used throughout the experiments.

### 2.2. Isolation and Screening of Bacterial Isolates for AgNPs Synthesis

Industrial effluent water sample was collected from a drain in Sahibabad Site-IV industrial area, U.P, India, in the month of December, 2017. The collected water sample was diluted serially and spread on Luria agar (LA) plates and overnight incubated at 37 °C. Then, morphologically distinct 12 isolates were streaked on LA plates to obtain pure colonies and named MS1 to MS12. All the isolates were screened for extracellular synthesis of AgNPs and based on their efficiency, isolate MS5 was selected and identified by 16S rRNA gene sequence analysis.

### 2.3. Biosynthesis of AgNPs

For the biosynthesis of AgNPs, the selected bacterial isolate was freshly inoculated in 250 mL Erlenmeyer flask containing 100 mL of Luria Broth (LB) and incubated at 37 °C at 120 rpm for 24 h. After incubation, the culture supernatant was obtained by centrifugation at 8000 rcf at 4 °C for 10 min and stored at 4 °C for further use. Cell-free culture supernatant was separately mixed with freshly prepared 1 mM AgNO_3_ solution at 1% (v/v) ratio in a reaction vessel and incubated at 40 °C. Culture supernatant at the same ratio without the addition of AgNO_3_ and only AgNO_3_ (1 mM) solution was used as control.

### 2.4. Characterization

Biosynthesis of AgNPs was monitored by visual observation of color change in the reaction mixture and measuring absorbance spectra in the scanning range of 300–800 nm by a double beam UV-Vis Spectrophotometer (Labtronics LT-2800, Haryana, India). Isolation and purification of AgNPs from solution were done by centrifugation at 8000 rcf at 4 °C for 30 min as described earlier [22]. Briefly, clear supernatant was discarded and pellet was washed four times by sterile Milli-Q water to remove the impurities. The isolated AgNPs were dried in hot air oven at 40 °C and powdered nanoparticles were used for characterization. The size and shape of purified AgNPs were studied by transmission electron microscope. For this, a drop of aqueous AgNPs solution was placed on carbon-coated copper grids and subsequently was dried at room temperature before transferring it to the microscope. Further, the morphology of particles was observed by FE-SEM (Zeiss Sigma) instrument equipped with energy-dispersive X-ray spectroscopy (EDX). The crystalline nature of particles was examined by X pertPro PANalytical X-ray diffractometer instrument using Cu-Kα radiation (k = 1.54 Å) operating at 45 kV with 40 mA. Fourier transform infrared spectroscopy (FTIR) spectrum analysis of powder AgNPs were recorded on a Bruker Tensor 37 instrument in the range of 4000–600 cm^−1^ at a resolution of 4 cm^−1^.

### 2.5. Isolation and Identification of Extended Spectrum β-Lactamase (ESBL) Producing Bacteria

Water samples were collected from Delhi stretch of river Yamuna, India to isolate ESBL producing bacteria. For this, serially diluted samples were spread on LA media plates and overnight incubated at 37 °C. After incubation, morphologically distinct colonies were picked from each plate and streaked on LA plates to obtain pure culture. All the pure bacterial isolates were screened by disc diffusion method using antibiotics belonging to third-generation cephalosporin to find out ESBL positive bacterial isolates using CLSI guidelines (Clinical and Laboratory Standards Institute, 2014). Those isolates showed zone of inhibition (ZOI) ≤ 22, ≤ 25, and ≤ 27 mm for the antibiotic ceftazidime, ceftriaxone, and cefotaxime, respectively, were suspected as ESBL positive and further screened by inhibitor potential disc diffusion (IPDD) test. In this IPDD test, third-generation cephalosporin used alone and with clavulanic acid, which is the major inhibitor of ESBL enzymes. Those isolates exhibited increase ZOI of ≥5 mm for the combination of antibiotic with clavulanic acid instead of only antibiotic were consider as positive for ESBL production. Finally, 16S rRNA gene sequence analysis technique was used to identify ESBL positive bacterial isolates, and nucleotide sequence data have been submitted to NCBI GenBank.

### 2.6. Antibiotic Profiling of ESBL Producing Bacteria

Antibiotic sensitivity test for ESBL positive isolates was done by disc diffusion as per Kirby Bauer’s method. For this, culture suspension was spread on Muller Hinton Agar (MHA) containing plates, and discs of standard antibiotics were placed at an appropriate distance and incubated overnight at 37 °C. The obtained ZOI (mm) results were interpreted as per the guidelines of Clinical and Laboratory Standards Institute, 2014. Antibiotics used in this study were amikacin (AK), ertapenem (ETP), imipenem (IMP), cefazolin (CZ), ampicillin (AMP), colistin (CL), polymyxin B (PB), rifampicin (RIF), trimethoprim (TR), and chloramphenicol (C).

### 2.7. Antibacterial Activity of AgNPs

Antibacterial properties of biosynthesized AgNPs were investigated against ESBLs producing bacterial isolates by well diffusion method. In brief, secondary culture suspension of tested bacterial isolates was spread on plates containing Mueller Hinton Agar media, and 6 mm size of diameter well was made by a sterile cork borer. Then, the AgNPs solution of different concentrations was prepared and poured 20 μL into each respective well, cell-free culture supernatant, and cefotaxime were used as control. Finally, all the plates were kept into an automated incubator overnight at 37 °C, and then ZOI occurred around the wells were measured in millimeters. The minimum inhibitory concentration (MIC) of AgNPs was done by the classical broth micro-dilution method in 96-well microtiter plates following the Clinical and Laboratory Standard Institute (CLSI, 2014) guidelines. Briefly, secondary culture of each isolate was prepared in LB medium and O.D of the cells were adjusted to 0.4 (~10^8^ CFU/mL) at 600 nm with the help of spectrophotometer and further diluted up to 10^6^ CFU/mL with same medium. The stock AgNPs solution (5.12 mg/mL) was prepared with sterile milli-Q water and sonicated for 30 min. Each well of microtiter plate was initially added with 100 µL of MHB medium and 80 µL of MHB further added into first row. Then, 20 µL of stock AgNPs solution (5.12 mg/mL) was added into the first row to achieve the final concentration of AgNPs (512 µg/mL). Then, two fold serial dilutions were prepared until row 10 to make a concentration gradient (512–1 µg/mL). Finally, 100 µL of test bacterial culture (10^6^ CFU/mL) was added into each well of the microtiter plate until row 11 and row 12 which were kept as control. The plates were sealed and kept for overnight incubation at 37 °C at 120 rpm. The lowest concentration of AgNPs inhibiting the growth of test bacterial isolate was considered as MIC, which was determined by a microplate reader. The experiment was carried out in duplicates in order to avoid any error.

The synergistic effects of AgNPs with four different classes of antibiotics were evaluated by disc diffusion method. For that, the antibiotic disc was impregnated with 20 μL of AgNPs (20 μg/disc) and overnight incubated at 37 °C to check their synergism. The antibiotics used in this experiment were ceftazidime (CAZ), ciprofloxacin (CIP), colistin (CL), and chloramphenicol (C).

### 2.8. Antifungal Activity of AgNPs

*Candida* isolates were obtained from the regional Sexually Transmitted Disease (STD) Centre, Safdarjung Hospital, New Delhi, India. All isolates were cultivated on yeast extract, peptone, and dextrose (YPD) medium (Himedia, India). Ethics clearance for sample collection was taken from the ethical committee Vardhman Mahavir Medical College and Safdarjung Hospital, New Delhi, India. The Minimum inhibitory concentration (MIC) of biosynthesized AgNPs against *Candida* spp. Including *C. albicans* 10261, *C. glabrata* 90, and *C. tropicalis* 985 was investigated by broth dilution method as previously described by Khan et al., 2011 [23]. Further, the growth curve experiment was performed to investigate the effect of AgNPs on the growth of *Candida* spp. For this, *Candida* spp. were grown in YEPD broth medium and adjusted O.D_595_ to 0.1 which approximately 10^6^ cells/mL and different concentrations of AgNPs added into individual culture flask, without AgNPs containing culture flask kept as a control. All the culture flasks were kept in a shaker incubator and growth of *Candida* spp. recorded at different time intervals using LaboMed Inc. Spectrophotometer (USA).

## 3. Results and Discussion

### 3.1. Isolation and Identification of the Bacteria

A total of twelve bacterial isolates collected from industrial effluent water were screened for the extracellular synthesis of AgNPs using their culture supernatant as reducing agent. To check their efficiency for extracellular synthesis of AgNPs, supernatant of each bacterial isolate was separately mixed with AgNO_3_ (1 mM) solution in a reaction tube and all tubes were incubated under the same experimental conditions. In order to identify the most efficient isolate, the color change of each reaction tube was monitored through visual observation. At first appearance of light brown color was observed in the MS5 reaction tube, indicated the rapid formation of AgNPs, as compared to other isolates. Further, an absorbance spectrum of each reaction mixture was taken by a double beam UV-Vis Spectrophotometer and analysis of data revealed that isolate MS5 was more efficient for maximum synthesis of AgNPs as compared to other isolates at same reaction time. Finally, bacterial isolate MS5 was identified by 16S rRNA gene sequence analysis. The obtained nucleotide sequence of 16S rRNA gene showed maximum homology with *Citrobacter* spp. in NCBI database. The sequence data of strain MS5 has been submitted to NCBI with an accession number MT044189.

### 3.2. Biosynthesis of AgNPs

**Figure 1:**
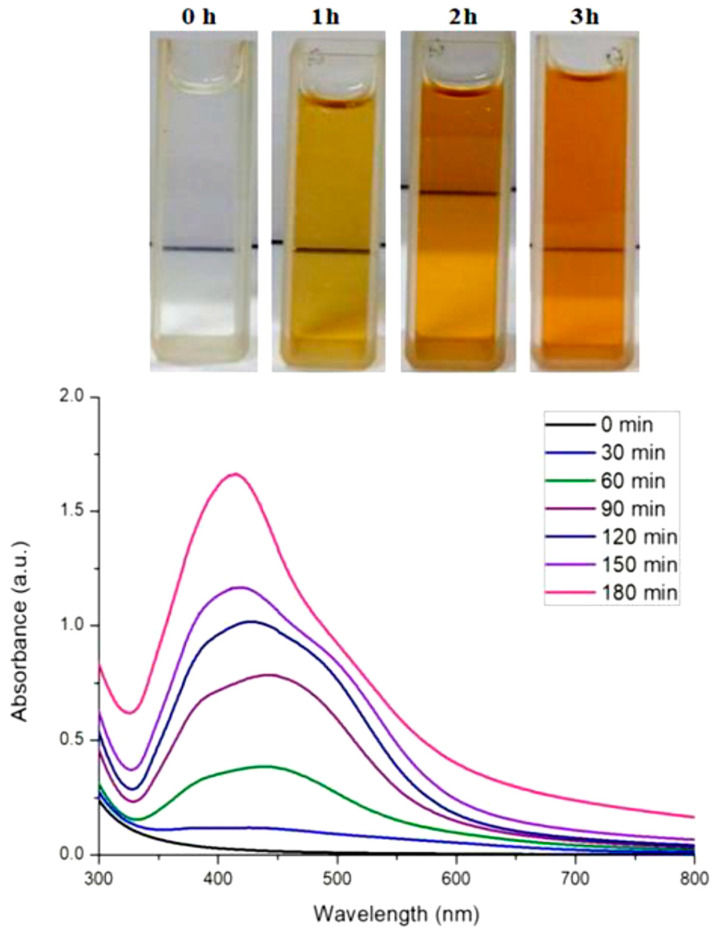
Showing the color change of reaction mixture from light to dark brown indicated the formation of AgNPs and UV-Vis spectra confirmed the time dependent biosynthesis of AgNPs.

Biosynthesis of AgNPs occurred when culture supernatant of *Citrobacter* spp. MS5 incubated with 1 mM AgNO_3_ solution at 40 °C. Initially, the formation of AgNPs was monitored through observing the changing of reaction mixture color, which turned light brown within 30 min and dark brown within 180 min of incubation (Figure 1). At the same time, there was no color change observer in the control conditions, which indicated that there was no formation of AgNPs. The reaction mixture turned to a brown color due to surface plasmon resonance (SPR) property of AgNPs indicated the formation of nanoparticles [24]. The observation of the present study is line with the previous reports where bacterial culture supernatant was used for biosynthesis of AgNPs [20,25].

### 3.3. Characterization

**Figure 2:**
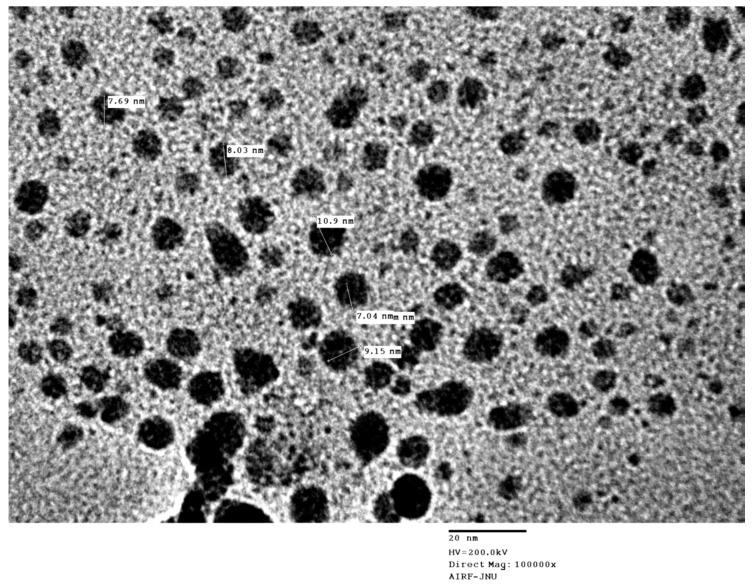
Transmission electron microscopy (TEM) image of biosynthesized AgNPs using *Citrobacter* spp. MS5 culture supernatant and AgNO_3_.

**Figure 3:**
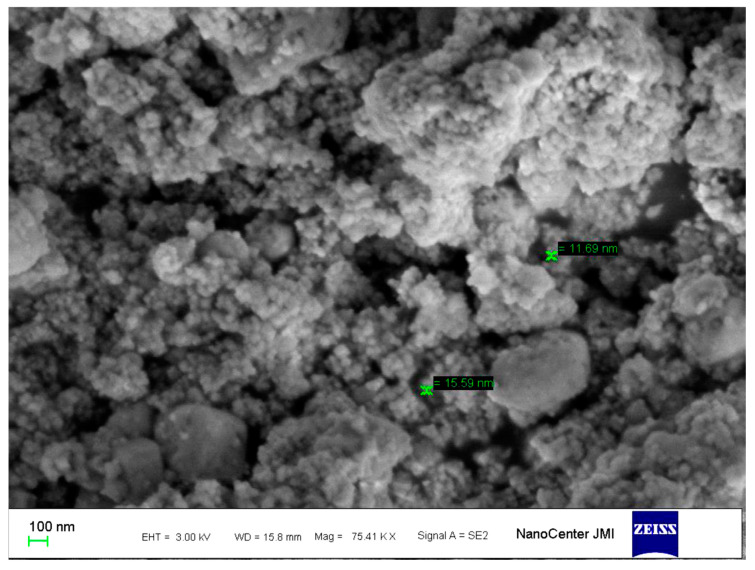
Scanning electron microscopy (SEM) image of biosynthesized AgNPs using *Citrobacter* spp. MS5 culture supernatant and AgNO_3_ (1 mM) solution.

**Figure 4:**
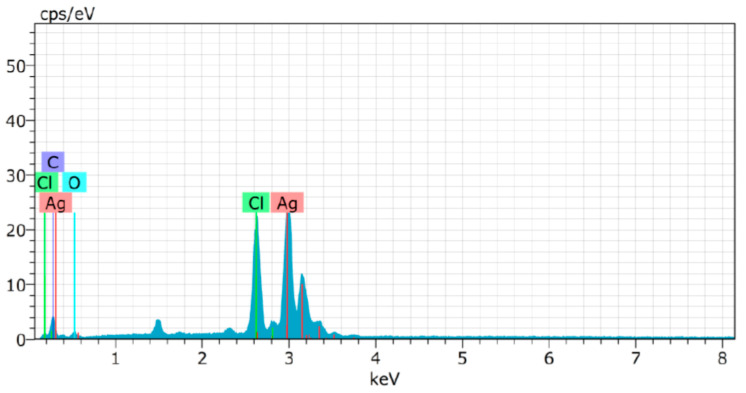
Energy dispersive X-ray (EDX) spectrum analysis of biosynthesized AgNPs showing strong peak at 3 keV for silver.

**Figure 5:**
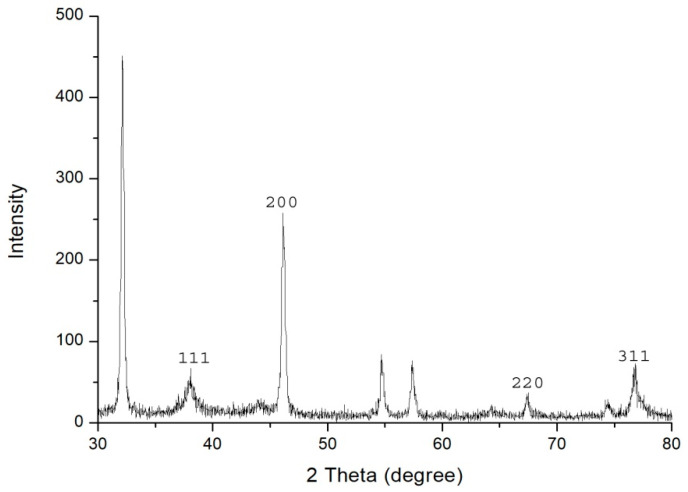
X-ray diffraction (XRD) pattern of biosynthesized AgNPs using culture supernatant of *Citrobacter* spp. MS5.

The UV-Vis spectral analysis were carried out at the regular time interval of 30 min to record the production of AgNPs are presented in Figure 1. The AgNPs formation increased with the progression of reaction time and maximum synthesis observed at 180 min, after that no significant change was observed in spectral characteristic. A strong absorbance band at 415 nm observed at 180 min of reaction due to (SPR) of nano-sized silver confirms the synthesis of AgNPs [26] (Figure 1). The analysis of transmission electron microscopy (TEM) images of biosynthesized AgNPs confirms that particles were spherical and size range of 5–15 nm (Figure 2). The morphology of AgNPs was further characterized by scanning electron microscopy (SEM) analysis, which also showed spherical shape, and the size has good agreement with TEM data (Figure 3). The elemental composition of biosynthesized AgNPs was investigated by EDX as shown in Figure 4. The EDX spectrum showed a strong peak for silver at 3 keV, which confirms the biosynthesis of metallic AgNPs [27,28]. Further, X-ray diffraction (XRD) analysis of AgNPs was carried out to determine their crystalline property and XRD pattern exhibited four distinct peaks at 2-theta values 38.39, 46.51, 64.76, and 77 corresponding to the intensities of (111), (200), (220), and (311) for the reflections of metallic silver (Figure 5). The data obtained are in good consistent with the database of Joint Committee on Powder Diffraction Standards (JCPDS file no. 04-0783). Although some extra peaks were observed in this spectrum of XRD, it may be due to the association of culture supernatant proteins with AgNPs during synthesis [22,28]. This kind of XRD pattern has been previously observed by various authors for extracellular biosynthesis of AgNPs [29,30].

**Figure 6:**
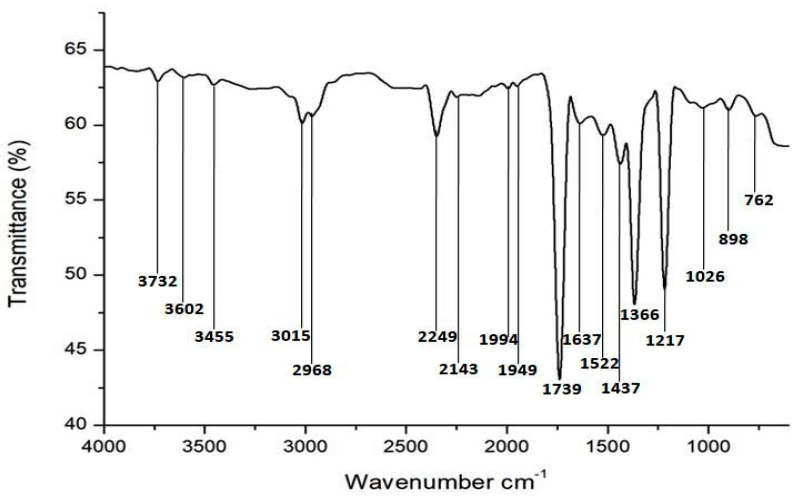
Fourier transform infrared spectroscopy (FTIR) spectrum of biosynthesized AgNPs using *Citrobacter* spp. MS5 culture supernatant as a reducing and stabilizing agent.

The possible role of bacterial culture supernatant biomolecules for biosynthesis as well as stabilizing AgNPs was determined by FTIR spectroscopy analysis. The FTIR spectrum showed major intense absorbance peaks at 3732 cm^−1^, 3602 cm^−1^, 3455 cm^−1^, 3015 cm^−1^, 2968 cm^−1^, 2249 cm^−1^, 2143 cm^−1^, 1994 cm^−1^, 1949 cm^−1^, 1739 cm^−1^, 1637 cm^−1^, 1522 cm^−1^, 1437 cm^−1^, 1366 cm^−1^, 1217 cm^−1^, 1026 cm^−1^, 898 cm^−1^ and 762 cm^−1^ (Figure 6). In this FTIR spectrum, some peaks located in the range of 3000–3600 cm^−1^ may be due to the presence of O-H and N-H groups with AgNPs [31,32]. The band located at 2249 cm^−1^ and 1739 cm^−1^ due to vibration of C-N and C=O groups respectively [33]. The peaks at 1637 cm^−1^ and 1522 cm^−1^ due to stretching vibration of NH groups represented the association of amide I and amide II of proteins molecules with AgNPs [34,35]. Further, the bands seen at 1366 cm^−1^, 1217 cm^−1^, and 1057 cm^−1^ may be due to the association of C-N in plane vibration of the aromatic and aliphatic amines, respectively [36]. The obtained FTIR data suggest that *Citrobacter* spp. MS5 culture supernatant biomolecules especially proteins play major role in the formation and capping of AgNPs [20,37]. The biosynthesis of AgNPs was only partially understood, as it was believed that nitrate reductase enzyme is mainly responsible for conversion of Ag^+^ to Ag^0^ and formation of AgNPs [28,38]. This enzyme also plays a major role for the conversion of nitrate to nitrite in the nitrogen cycles [39].

### 3.4. Isolation, Identification, and Antibiotic Profiling of ESBL Producing Bacteria

All the ESBL positive bacteria were isolated from Delhi stretch of river Yamuna, India, and identified as *K. pneumoniae* BK4, *E. coli* SK30, *Enterobacter hormaechei* NK15 by 16S rRNA sequences data analysis. The 16S rRNA nucleotide gene sequences have been submitted to NCBI GeneBank with the accession no. of KY471704, MT044305, and MT044306 for *K. pneumoniae* BK4, *E. coli* SK30, and *Enterobacter hormaechei* NK15, respectively. The emergence of ESBL producing Gram-negative bacteria in natural as well as clinical settings creates a global problem for human health. Our previous study reported the occurrence of multidrug-resistant ESBL positive bacteria from river Yamuna, India [9]. The ESBL producing bacteria can easily reach up to drinking water as well as human food chain is a major concern [40]. Antibiotic susceptibility of all ESBL producing Gram-negative bacteria towards seven different classes of antibiotics was analyzed as per CLSI guidelines and isolates characterized as sensitive or resistant. All the tested bacteria have resistance for the antibiotic AMP, CAZ, TR, and sensitivity for AK. The isolates considered multidrug-resistant (MDR) which have resistance against at least three different classes of antibiotics [41]. Analysis of antibiotic profiling data revealed that all ESBL producing bacterial isolates have MDR phenotype.

### 3.5. Antibacterial Activity of AgNPs

**Figure 7:**
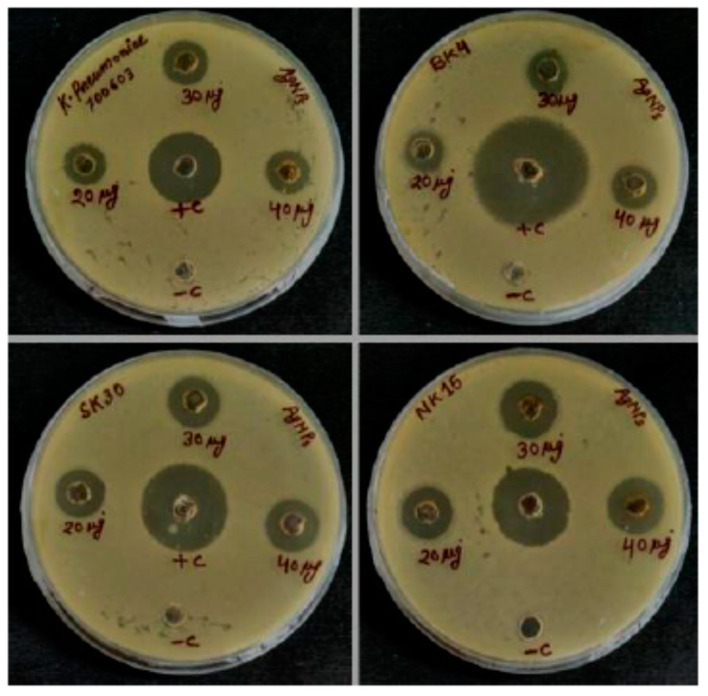
Antibacterial activity of biosynthesized AgNPs at different concentration against extended spectrum β-lactamase (ESBL) producing *K. pneumoniae* 700603, *K. pneumoniae* BK4, *E. coli* SK30, and *Enterobacter hormaechei* NK15 by well diffusion method.

Antibacterial activity of biosynthesized AgNPs was investigated against ESBL positive Gram-negative isolates by well diffusion method. Clear ZOI was observed across the wells on culture loaded MHA plates towards all test isolates are shown in Figure 7. The increasing ZOI was observed as concentration of AgNPs increase and maximum activity recoded at 40 µg (Table 1). No antibacterial activity was observed for the culture supernatant (used as negative control). The highest and lowest antibacterial activity of AgNPs in contrast to ZOI was observed against *Enterobacter hormaechei* and *K. pneumoniae*, respectively. The MIC values of AgNPs against all test isolates were determined by the broth dilution method, and minimum concentration responsible to stop bacterial growth was considered as MIC. The MIC values for all tested isolates varied from 4–8 µg/mL are represented in Table 1. The previous studies also reported the antibacterial activity of AgNPs against ESBL producing bacteria and our results have a correlation with them [5,6]. The antibacterial activities of antibiotics with AgNPs combinations were more than antibiotics alone against all tested ESBL positive Gram-negative bacteria. The diameter of zone of inhibition (ZOI) for different antibiotic discs with and without AgNPs is shown in Table 2. The increase in fold area is the ZOI around the discs due to cumulative effects of AgNPs in combination with different antibiotics compared to antibiotic alone. The overall synergistic effect is the average value of increase in fold area for a particular antibiotic in combination with AgNPs against four tested bacterial isolates is represented in Table 2. The highest overall fold increase was observed for AgNPs in combination with the antibiotic ceftazidime (CAZ) followed by colistin (CL), ciprofloxacin (CIP), and chloramphenicol (C) against all isolates (Table 2). Typically, the maximum fold increase was observed in *Enterobacter hormaechei* and *K. pneumoniae* for the combination of CAZ+AgNPs and *E. coli* for CL+AgNPs combination. Some previous reports also found the effective combined antibacterial activity of AgNPs with standard antibiotics against drug-resistant bacteria [42,43].

### 3.6. Antifungal Activity of AgNPs

**Figure 8:**
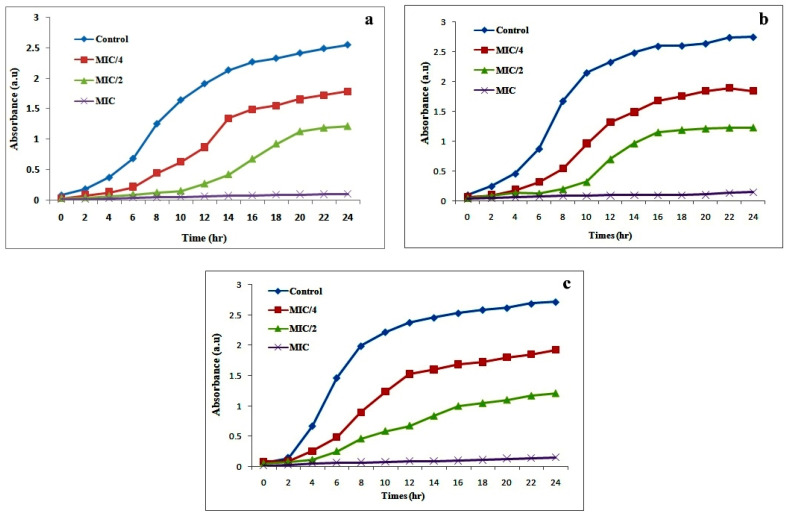
Growth curves pattern of *C. albicans* (**a**), *C. glabrata* (**b**), and *C. tropicalis* (**c**) in the presence of different concentration of biosynthesized AgNPs.

Minimum inhibition concentration (MIC) values of AgNPs against three different *Candida* strains are given in Table 3. Biosynthesized AgNPs showed the lowest MIC values for *C. albicans* 10261 and higher for both *C. glabrata* 90 as well as *C. tropicalis* 985. These different MIC values may be because of pleomorphic properties and variation of cell walls structural organization among *Candida* spp. [44]. The growth pattern of tested *Candida* spp. in the presence and absence of AgNPs was monitored up to 24 h is shown in Figure 8. The normal growth pattern with distinct lag, log, and stationary phase was observed for all untreated isolates. Concentration-dependent antifungal activity of AgNPs was recorded against all tested isolates and their growth pattern was differed as compared to untreated cells (Figure 8). At MIC concentration, the complete inhibition of growth and no distinct growth phases was observed for all tested *Candida* strains. The increased lag phase was noted at sub MIC concentration of AgNPs. The short log phase, earlier lag phase, and concentration-dependent suppressed stationary phase were witnessed against all tested *Candida* strains as compared to control cells. The previous studies also reported that AgNPs can affect the growth of *Candida* cells and we observed a similar kind of growth pattern [45,46].

Several studies reported the antibacterial activity of AgNPs but their exact mechanisms of action are yet to be established. According to various previous reports, AgNPs could get interact with bacterial outer lipopolysaccharides, cell wall, membrane, and disrupt them [47]. AgNPs also enter into the bacterial cell and bind with thiol group of sulfur-containing proteins, enzymes, and DNA, leading to cell death [4,48]. Combined antibacterial activity of AgNPs with different classes of antibiotics may be due to bonding reaction between functional groups of antibiotics with AgNPs by chelation [37]. The antifungal activity of AgNPs, due to their interaction with the fungal cell wall and membrane, disrupts the integrity due to the formation of pores, leading to lysis of cell [49]. It was also thought that AgNPs enter into the fungal cell and bind with function (-SH) groups of protein, DNA, and disrupt the enzymatic activity, produced reactive oxygen species, which leads to cell death [47,50]. This considerable antibacterial and antifungal activity of AgNPs need further study to explore their exact target sites as well as mode of action in order to develop new therapy to combat ESBLs producing multidrug-resistant bacteria and *Candida* spp.

## 4. Conclusions

The present study describes an easy and eco-friendly extracellular synthesis approach of AgNPs using environmental bacterial isolate of *Citrobacter* spp. MS5 culture supernatant as reducing and stabilizing agent. To the best of our knowledge, biosynthesis of AgNPs using this isolate has not been reported so far. The involvement of bacterial culture supernatant biomolecules during the formation and capping of AgNPs has demonstrated by FTIR analysis. Biosynthesized AgNPs are metallic, crystalline, and spherical in shape having a size range of 5–15 nm. Further, biosynthesized AgNPs revealed good antimicrobial activity against ESBL producing multidrug-resistant Gram-negative bacteria. Additionally, it also exhibits enhanced antibacterial activity in combination with different antibiotics against all tested bacterial isolates. Further, effective antifungal activity of biosynthesized AgNPs was observed against pathogenic *Candida* spp. The results of the present study suggests that bacteria isolate *Citrobacter* spp. MS5 may be used for rapid mass synthesis of AgNPs and biosynthesized AgNPs could be used as an alternative to treat ESBL producing MDR bacteria as well as *Candida* spp. infections, but required further in vivo study.

## Figures and Tables

**Table 1:** Antibacterial activity of biosynthesized AgNPs against various ESBL producing Gram-negative bacteria by well diffusion method.

Bacterial Strains	ZOI (mm) for Different Concentration of AgNPs					MIC (µg/mL)
	20 µg	30 µg	40 µg	+ ve Con.	− ve Con.	
*K. pneumoniae* 700603	11	12	12	20	0	8
*K. pneumoniae* BK4	11	12	13	30	0	8
*E. coli* SK30	12	13	14	23	0	4
*Enterobacter hormaechei* NK15	13	15	15	21	0	4

**Table 2:** The zone of inhibition (mm) of different antibiotics alone and in combination with AgNPs against ESBL producing bacteria by disc diffusion method.

Antibiotics	*K. pneumoniae* 700603	*K. pneumoniae* BK4	*E. coli* SK30	*Enterobacter hormaechei NK*15	Over All Synergistic Effect
A. CAZ AgNPs B. CAZ + AgNPs Increase in fold area *	8 11 10 0.56	11 11 14 0.61	8 12 10 0.56	6 13 11 2.36	**1.0**
A. CIP (5 µg) AgNPs B. CIP + AgNPs **Increase in fold area**	25 11 27 0.16	25 11 27 0.16	20 12 22 0.21	11 13 13 0.39	**0.23**
A. CL AgNPs B. CL + AgNPs **Increase in fold area**	16 11 16 0	12 11 15 0.56	12 12 16 0.77	12 13 15 0.56	**0.47**
A. C (30 µg) AgNPs B. C + AgNPs **Increase in fold area**	20 11 21 0.10	24 11 27 0.26	26 12 28 0.16	27 13 28 0.07	**0.14**

* Fold increases for different antibiotics were calculated by the formula (B^2^ − A^2^)/A^2^, where A and B are the zone of inhibition for only antibiotic and antibiotic with AgNPs, respectively. In case of no zone of inhibition, diameter of the disc (6 mm) was considered for the calculation.

**Table 3:** Antifungal activity (MIC) of biosynthesized AgNPs against different *Candida* strains.

*Candida* Strains	Flucanazol (µg/mL)	AgNPs (µg/mL)
*C. albicans* 10261	16	100
*C. glabrata* 90	16	150
*C. tropicalis* 985	>64	150
